# The Use of Baths in the Summer Complaint of Children

**Published:** 1875-10

**Authors:** J. G. Thomas


The Use of Baths in the Summer-Complaint of Children.—*By J. G. Thomas, M.D.*—This disease has been almost epidemic with us this summer, and accompanied with more persistent and obstinate fever than I have ever before known it.

The treatment alone by internal means has been very unsatisfactory; in fact, with me it has always been so in a certain percentage of the cases, for the reason that it is oftentimes impossible to get medicines to remain on the stomach.

In the cases coming under my observation the temperature ranged from 101° to 105°; and it was those of a very high degree of fever that caused me to get in the habit of using what I call the *reduction* treatment by cold water; and I now employ it in all cases of any fever, varying the temperature by the temperature of the patient. Usually by placing a little patient with fever of 102° to 103° or 104°, in water of temperature 70° to 85° for twenty or thirty minutes, the heat will run down to the normal point or below it, and the child will go quietly to sleep, and wake up in an hour or two, much improved. In families where I have used this for the first time, I have found it prudent to stay for an hour or two and see the treatment properly carried out, for many nurses and mothers will at first be afraid of it, but they will soon get over all this fear when they observe the charming effect it has upon the child. * * *

I first take the temperature of the child before it goes in the bath, and then the temperature of the bath, and, as a rule, have the child put into the water at the supposed temperature at which it is in the habit of bathing. But some children are very nervous about going into water: with such as these we should be very deliberate, and should allow them to get accustomed to it. They should be put into water a little above tepid, to which colder water should be gradually added until the temperature is reduced to 75° ; or, when the fever is very hot, and does not yield readily, to 70°. In many cases the children will scream a short time, but if they are properly managed their cries soon cease, and they wake up an hour after they are taken out, much improved ; whereas before, they were unable to sleep for the nervous twitchings and threatenings towards convulsions. The child, after it has been in the bath for from twenty minutes to half an hour, should be taken out, the axillary region dried with a soft towel, and its temperature taken. If we find the heat has gone down to the normal degree or a little below, the child should be wrapped in a light woolen blanket and put in its bed, when, as said above, it will almost invariably sleep for some time. But in one hour its temperature should be taken again, and if it has gone up, the same process should be gone over. Thus I have kept it up in some persistent and severe cases for days, making the child comfortable, and giving time for the effect of other controlling remedies.

In some cases the fever once reduced in this way will not return for four, five, six, or twelve hours, whilst in others it does not return at all ; and in all the baths control the heat so as to make the case one of much less gravity, provided they be properly and persistently used.

I have persuaded myself, since the introduction of the clinical thermometer into use, that heat in the blood sometimes causes convulsions, and that there are many cases in scarlatina, for instance, as well as other diseases, which have died from the effect of heat upon the globules of the blood, which we are in the habit of attributing to the effect of poison of the disease. This conclusion of course is founded alone upon clinical observation, without any pathological investigation. * * *

I have also tried the same reduction treatment in a few cases of malarial fever this season in adults ; in these it has acted in the same happy way.

There are scattering cases reported in the journals where it has been tried in rheumatism, scarlatina, etc., with high degrees of temperature, in all of which I propose to try it in future, particularly in scarlatina where the eruption does not appear upon the surface as it should, and the temperature of the patient ranges high.—*Philadelphia Medical Times.*

